# Sex differences in endurance exercise capacity and skeletal muscle lipid metabolism in mice

**DOI:** 10.14814/phy2.15174

**Published:** 2022-02-08

**Authors:** Lola E. Holcomb, Patrick Rowe, Caitlin C. O’Neill, Elizabeth A. DeWitt, Stephen C. Kolwicz

**Affiliations:** ^1^ Heart and Muscle Metabolism Laboratory Department of Health and Exercise Physiology Ursinus College Collegeville Pennsylvania USA

**Keywords:** exercise metabolism, exercise physiology, fatty acid oxidation, ketosis, triglyceride metabolism

## Abstract

Previous studies suggest that sex differences in lipid metabolism exist with females demonstrating a higher utilization of lipids during exercise, which is mediated partly by increased utilization of muscle triglycerides. However, whether these changes in lipid metabolism contribute directly to endurance exercise performance is unclear. Therefore, the objective of this study was to investigate the contribution of exercise substrate metabolism to sex differences in endurance exercise capacity (EEC) in mice. Male and female C57BL/6‐NCrl mice were subjected to an EEC test until exhaustion on a motorized treadmill. The treadmill was set at a 10% incline, and the speed gradually increased from 10.2 m/min to 22.2 m/min at fixed intervals for up to 2.5 h. Tissues and blood were harvested in mice immediately following the EEC. A cohort of sedentary, non‐exercised male and female mice were used as controls. Females outperformed males by ~25% on the EEC. Serum levels of both fatty acids and ketone bodies were ~50% higher in females at the end of the EEC. In sedentary female mice, skeletal muscle triglyceride content was significantly greater compared to sedentary males. Gene expression analysis demonstrated that genes involved in skeletal muscle fatty acid oxidation were significantly higher in females with no changes in genes associated with glucose uptake or ketone body oxidation. The findings suggest that female mice have a higher endurance exercise capacity and a greater ability to mobilize and utilize fatty acids for energy.

## Graphical abstract

The findings of the study suggest that female mice have a higher endurance exercise capacity due to an increased ability to mobilize fatty acids and utilize both exogenous and endogenous fatty acids for energy within the skeletal muscle.

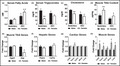

## 1 INTRODUCTION

The consideration of sex as a biological variable has become an important factor in experimental study designs (Clayton, 2018). Since female subjects are under‐represented in biomedical and clinical research (Costello et al., 2014; Vitale et al., 2017), there are likely significant deficiencies in the understanding of sex‐specific differences in physiology and metabolism, which may have profound consequences on human health and performance. Therefore, additional research studies focused on the identification and characterization of differences in biological, physiological, and metabolic processes between males and females are needed.

Sex differences in exercise and sports performance are well‐documented. Males are reported to have greater measures of muscular strength (Miller et al., 1993) and maximal volume of oxygen consumption (VO_2max_) (Joyner, 1993). However, females are suggested to have reduced performance fatigability, which is also referred to as muscle fatigue (Hunter, 2016). Therefore, females may exhibit a greater endurance exercise capacity than males. Few animal studies have directly addressed this question of sex differences in endurance capacity (Konhilas et al., 2004; McMullan et al., 2016; Oydanich et al., 2019; Sun et al., 2018). Although the findings support the notion that female mice have greater endurance exercise performance than males, these studies generally rely on voluntary wheel running (Konhilas et al., 2004; McMullan et al., 2016) or exercise bouts of shorter duration (~30 min) (Konhilas et al., 2004; Oydanich et al., 2019). Given the potential sex differences that exist in substrate metabolism, additional studies evaluating endurance exercise capacity, particularly of longer durations, are warranted.

Past research identified various sex differences in substrate metabolism, particularly related to lipid metabolism. Respiratory exchange ratio (RER) has been shown to be lower in females during endurance exercise, which would indicate a greater utilization of lipids over carbohydrates (Carter et al., 2001; Horton et al., 1985). Females also may have greater release and uptake of fatty acids in skeletal muscle during endurance exercise (Mittendorfer et al., 2002). Females have been reported to have higher basal skeletal muscle triglyceride content, which could lead to greater utilization of endogenous lipids during endurance exercise (Roepstorff et al., 2002; Roepstorff et al., 2006a; Steffensen et al., 2002). These reported changes in lipid metabolism were identified in aerobic exercise of constant load or submaximal intensity at fixed duration. However, whether these enhancements in lipid metabolism contribute directly to higher endurance exercise capacity in females is not clear.

The purpose of the present study was to determine whether changes in lipid metabolism were responsible for enhanced endurance exercise capacity in female mice. To test this, we compared endurance exercise capacity in male and female mice using a motorized treadmill. The testing protocol lasted up to 2.5 h, to specifically target the interaction of lipid metabolism and endurance exercise since the utilization of lipids becomes more predominant during longer durations of moderate intensity in both mice and humans (Egan & Zierath, 2013; Faldt et al., 2004). We performed biochemical analyses on serum and tissues harvested at the immediate conclusion of the exercise session to determine the contribution of lipids to exercise performance. Finally, we analyzed gene expression of heart, liver, and skeletal muscle tissues to identify potential sex differences in glucose, fatty acid, and ketone body metabolism on a molecular and genetic scale.

## 2 MATERIALS AND METHODS

### 2.1 Animal model

All experimental protocols used in this study were approved by the Institutional Animal Care and Use Committee (IACUC) of Ursinus College. Male (*n* = 18) and female (*n* = 23) C57BL/6‐NCrl mice were obtained from an in‐house colony from breeders obtained from Charles River Laboratories. A cohort of male and female mice (*n* = 9 each group) were assigned to participate in an endurance exercise capacity (EEC) test. An additional cohort of male (*n* = 9) and female (*n* = 14) mice that did not participate in the exercise test were used as sedentary controls. All mice were 4–5 months of age. Mice were maintained in an animal care facility that followed a 12‐h light/dark cycle. All mice were fed standard rodent chow (Lab Diet, #5001). Food and water were provided ad libitum.

### 2.2 Endurance exercise capacity test

Male and female mice were subjected to an endurance exercise capacity (EEC) test on a motorized treadmill following a protocol adapted from a previous study (Beylot et al., 2012). The EEC protocol consisted of a graded test with increasing speeds for up to 2.5 h until exhaustion. The treadmill grade was kept constant at 10%. Speeds were adjusted using the following protocol: 10.2 m/min for 30 min, 12.6 m/min for 15 min, 15.0 m/min for 15 min, 17.4 m/min for 15 min, 19.8 m/min for 15 min, and 22.2 m/min for up to 60 min. Mice were constantly monitored and were motivated to exercise via gentle prodding of the hindquarters (Poole et al., 2020). The EEC was discontinued when the mice were unable to run continuously on the treadmill for at least 10 s or the final stage was completed. Prior to the EEC, all mice were acclimated on the treadmill for 4 consecutive days for 10 min at a speed of 10 m/min. The mice were exercised near or at the start of the dark cycle.

### 2.3 Tissue and blood harvest

Immediately at the end of exercise, mice that participated in the EEC received an intraperitoneal (IP) injection of ~170 mg/kg of sodium pentobarbital. After confirming sedation with a toe‐pinch test, the heart was removed, rinsed in ice cold phosphate buffered saline, trimmed of excess tissue, blotted dry, and frozen in liquid nitrogen. Blood was collected from the chest cavity. A portion of the liver and a section of the gastrocnemius muscle was dissected, blotted dry, and frozen. An identical procedure for the collection of tissue and blood was followed for mice in the sedentary groups with the exception that sedentary mice were fasted for approximately 2 h. Blood was allowed to clot on ice and then centrifuged at 2000g for 20 min at 4°C. The serum was removed and transferred to a new tube. All tissues and serum were stored at −80°C until additional analyses were performed.

### 2.4 Serum analysis

Blood obtained from the mouse tail tip immediately at the end of exercise and prior to anesthesia was used to measure blood glucose using a hand‐held glucometer (Contour, Ascensia Diabetes Care, Parsippany, NJ). Ketone bodies (acetoacetate and 3‐hydroxybutyrate), non‐esterified fatty acids (NEFA), triglycerides, and total cholesterol were measured in serum harvested from the chest cavity using commercially available colorimetric kits (FUJIFILM Wako Diagnostics U.S.A., Mountain View, CA) per manufacturer's instructions. Serum concentrations of 17‐beta estradiol was quantified by a commercially available ELISA kit (#108667, Abcam, Waltham, MA). Serum lactate was measured using a commercially available reagent (#735‐10, Trinity Biotech, Wicklow, Ireland). Absorbance values were measured on a spectrophotometer (Smartreader 96, Accuris Instruments, Edison, NJ). Concentrations were determined from a calibration curve with commercially available standards obtained from the manufacturer.

### 2.5 Biochemical analysis in tissues

Glycogen content was assessed in frozen liver and gastrocnemius tissues by determining the amount of glucose released from glycogen with a commercially available kit (GAHK‐20, Sigma Aldrich, St. Louis, MO). Glycogen was separated from exogenous glucose in the tissue using an alkaline extraction procedure (Passonneau & Lauderdale, 1974). Triglycerides were extracted and measured from frozen gastrocnemius using a triglyceride colorimetric assay kit (#10010303, Cayman Chemicals, Ann Arbor, MI).

### 2.6 RNA isolation and gene expression analysis

RNA isolation and Real Time Polymerase Chain Reaction (RT‐PCR) was performed as previously reported (Choi et al., 2016; Ritterhoff et al., 2021). In brief, RNA was extracted from the heart, quadriceps, and liver tissues using the RNeasy Mini Kit (#74104, Qiagen, Hilden, Germany). cDNA synthesis was performed using Omniscript reverse transcriptase (RT) kit and random primers according to the manufacturer's instructions (#205111, Qiagen, Hilden, Germany). RT‐PCR was performed using SYBR‐Green (Bio‐Rad, Hercules, CA) and cDNA transcripts were quantified using a relative standard curve. The calculated values for each gene were then normalized to a housekeeping gene (18S rRNA). Values obtained from the RT‐PCR analysis are reported as fold change over sedentary males.

### 2.7 Statistical analysis

All data are presented as means ± standard error of mean (SEM). For two group comparisons, statistical analysis was tested using a Student's *t*‐test. For four group comparisons, a two‐way analysis of variance (ANOVA) with Tukey's post‐hoc analysis was used to test the effect of sex and exercise on dependent variables of serum measures and tissue content. Statistical significance was tested at the *p* < 0.05 level. All graphs were created and analyses were performed using GraphPad Prism 9.0 (GraphPad Software, San Diego, CA).

## 3 RESULTS

### 3.1 Female mice have greater endurance exercise capacity

**FIGURE 1:**
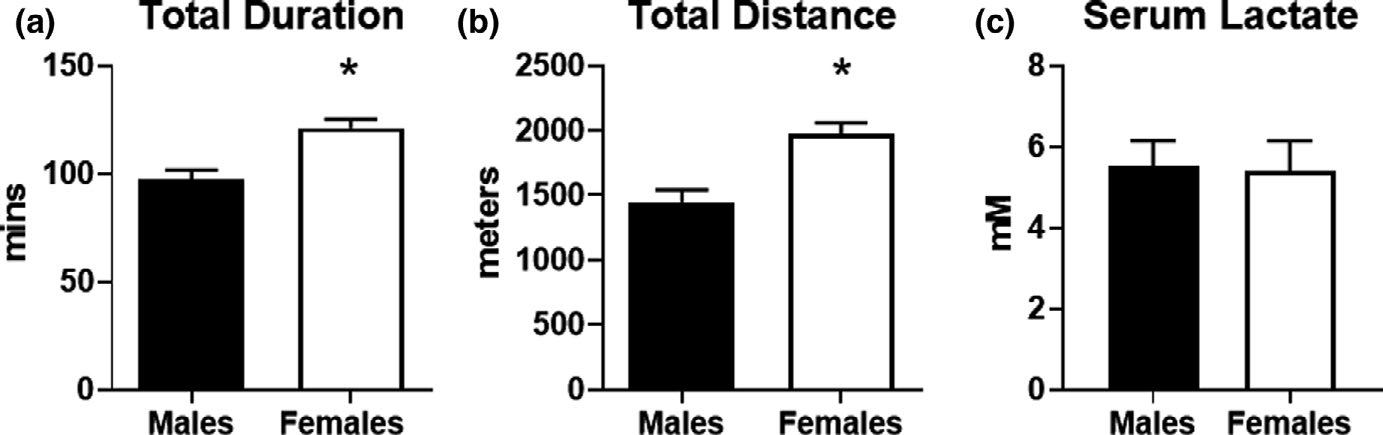
Assessment of endurance exercise capacity in male and female mice. Mice were subjected to an endurance exercise capacity (EEC) test on a motorized treadmill. Incline was set to 10% and speeds were adjusted at regular intervals for up to 2 h. Exercise performance was assessed in male and female mice (*n* = 9 each group) by (a) total duration in minutes and (b) total distance in meters. (c) Serum lactate was measured in serum obtained from blood of male and female mice collected immediately at the conclusion of the EEC test (*n* = 5 each group). *p < 0.05 vs. males

To assess endurance exercise capacity, male and female mice were subjected to a graded exercise test on a motorized treadmill. We selected an exercise test protocol that increased intensity after longer intervals to ensure achievement of steady state metabolism and allow the mice to exercise for up to 2.5 h. As shown in Figure 1a, female mice exhibited endurance exercise times that were approximately 25% higher than male mice. In accordance with this finding, female mice also ran farther distances than did males (Figure 1b). Since blood lactate levels are associated with exercise intensity, we measured serum lactate from blood harvested from mice immediately at the end of exercise. Males and females had equivalent lactate levels (Figure 1c). These data demonstrate that female mice have a higher endurance exercise capacity than male mice.

### 3.2 Relative heart and skeletal muscle mass are similar in males and females

**FIGURE 2:**
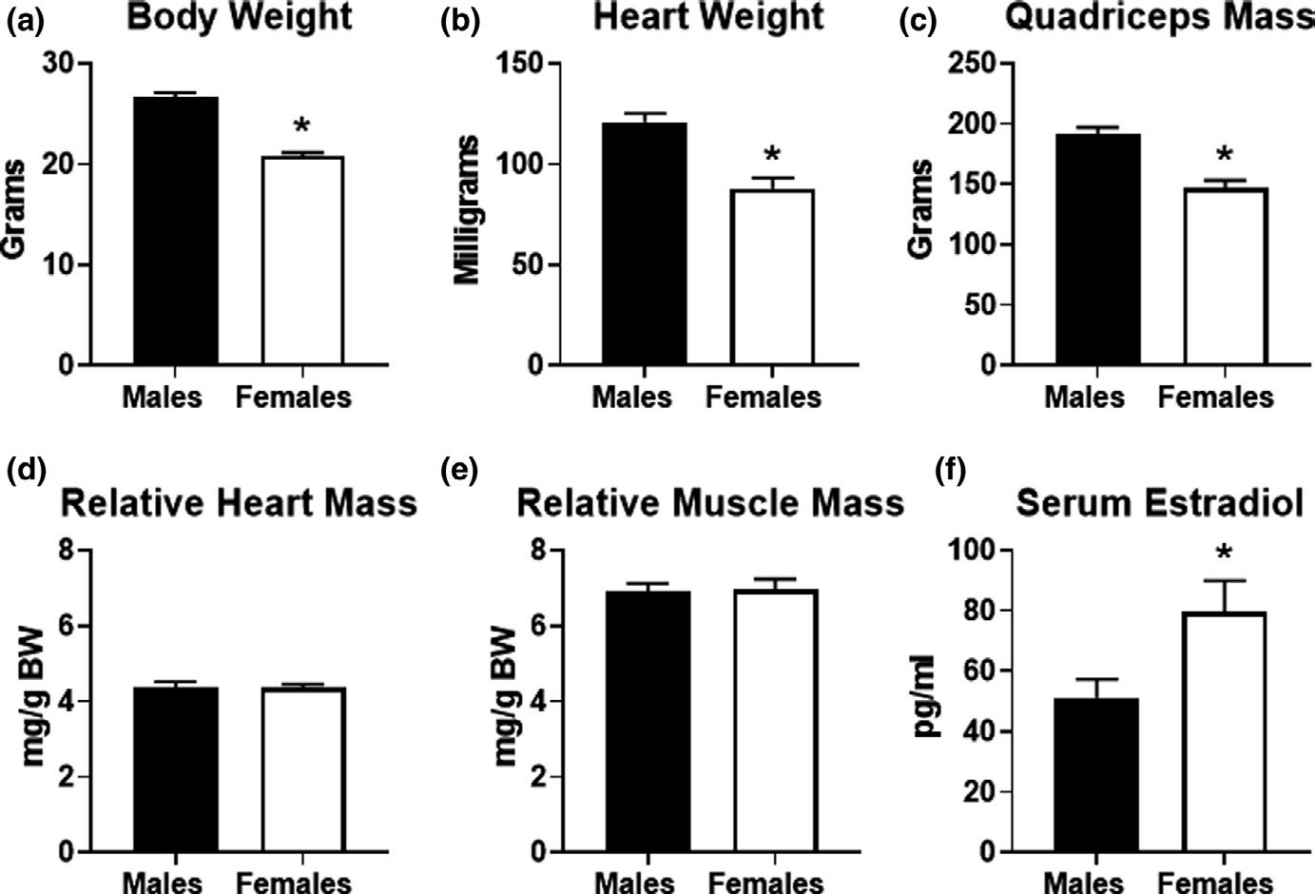
Physical characteristics of male and female mice. (a) Body weight (BW) obtained in male (*n* = 18) and female (*n* = 23) mice that participated in the study. (b) Heart weight, (c) quadriceps mass, (d) heart weight to BW and (e) quadriceps to BW ratios in male (*n* = 9) and female (*n* = 14) sedentary (non‐exercised) mice. (f) 17‐beta‐estradiol levels measured in the serum obtained from a random sampling of male and female mice (*n* = 5 each group). *p < 0.05 vs. males

Physical differences in males and females are common, which could account for differences in exercise performance. As expected, body weight, heart weight, and quadriceps mass were all significantly lower in female mice compared to males (Figure 2a–c). The lower body weight in females could offer an advantage in exercise performance since the overall weight load is less. However, the lower absolute muscle mass in cardiac and skeletal mass in female mice might also provide a disadvantage in exercise performance. Therefore, we calculated relative heart mass and quadriceps mass ratios. When heart or quadriceps mass was normalized to body weight, there were no significant differences in female mice (Figure 2d, e). We also measured 17‐beta estradiol levels in the serum from a cohort of mice. As expected, serum estradiol levels were significantly higher in female mice (Figure 2f). Overall, these findings suggest that body weight and/or muscle mass are not likely to contribute significantly to the sex‐specific differences in endurance exercise capacity in mice.

### 3.3 Measures of glucose metabolism during endurance exercise is similar in males and females

**FIGURE 3:**
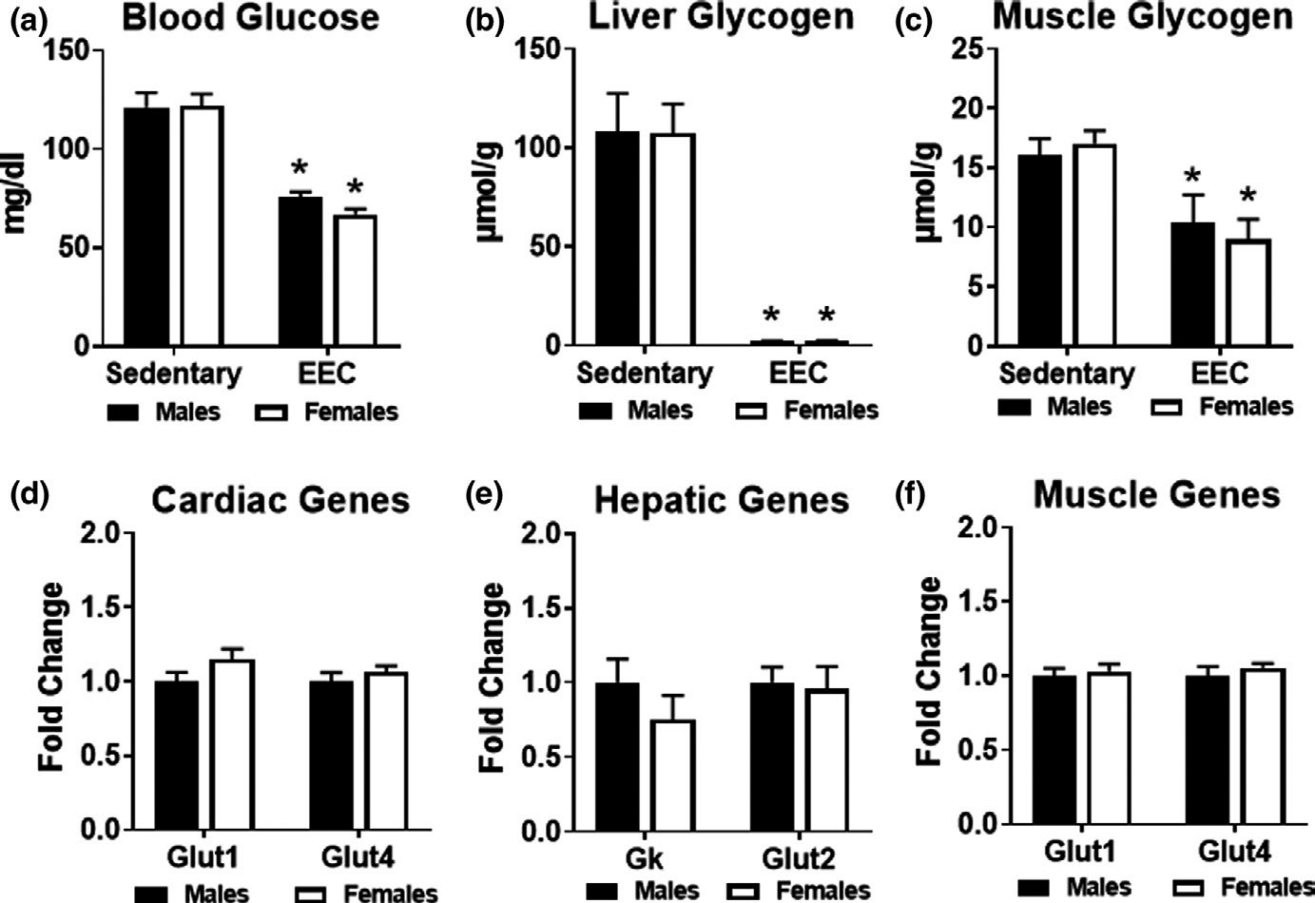
Analysis of glucose metabolism in male and female mice. (a) Blood glucose measured in male and female mice at the immediate conclusion of the endurance exercise capacity (EEC) test (*n* = 9 each group). A cohort of sedentary (non‐exercised) male (*n* = 9) and female (*n* = 14) mice were used as controls. Two‐way ANOVA revealed a significant main effect of exercise on blood glucose (F(1,38) = 76.69, *p* < 0.0001). (b) Glycogen content measured in liver obtained from sedentary and EEC male and female mice (*n* = 5–7 each group). Two‐way ANOVA revealed a significant main effect of exercise on liver glycogen content (F(1,18) = 72.20, *p* < 0.0001). (c) Glycogen content measured in gastrocnemius muscle obtained from sedentary and EEC male and female mice (*n* = 5–7 each group). Two‐way ANOVA revealed a significant main effect of exercise on gastrocnemius glycogen content (F(1,24) = 16.17, *p* = 0.005). Gene expression analysis of glucose metabolism in (d) Cardiac, (e) Hepatic and (f) Quadriceps muscle tissue harvested from sedentary (non‐exercised) male and female mice (*n* = 4–5 each group). *Glut1*, glucose transporter 1; *Glut4*, glucose transporter 4; *Gk*, glucokinase; *Glut2*, glucose transporter 2. *p < 0.05 vs. male or female sedentary group

To gain insight into the use of glucose as a fuel during exercise, we compared blood glucose levels and glycogen content of liver and gastrocnemius muscles in sedentary and exercised mice. Male and female mice exhibited significantly lower blood glucose levels at the end of exercise when compared to sedentary controls (Figure 3a). As shown in Figure 3b, liver glycogen content was significantly depleted to an equal extent in males and females at the end of the exercise test. In addition, muscle glycogen content was significantly lower in exercised males and females compared to sedentary counterparts, with no significant difference between the two groups (Figure 3c). We analyzed gene expression in the heart, liver, and quadriceps muscle in sedentary male and female mice to detect baseline differences in genes related to glucose metabolism. Expression of the glucose transporters, *Glut1*, *Glut2*, *Glut4*, were similar in the liver, heart, and muscle of male and female mice (Figure 3d–f). Overall, these data suggest that basal glucose metabolism is similar in males and females. Furthermore, males and females demonstrate similar usage patterns of exogenous and endogenous glucose during endurance exercise.

### 3.4 Exercise‐induced ketosis is greater in female mice during endurance exercise

**FIGURE 4:**
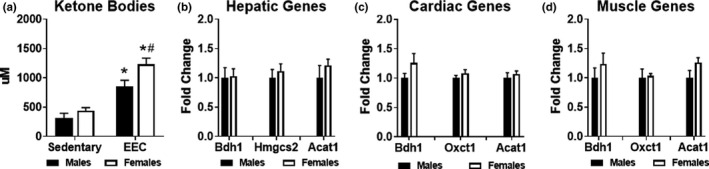
Evaluation of ketone body metabolism in male and female mice. (a) Serum ketone bodies (acetoacetate and 3‐hydroxybutyrate) measured in male and female mice at the immediate conclusion of the endurance exercise capacity (EEC) test (*n* = 9 each group). Serum obtained from sedentary (non‐exercised) male (*n* = 9) and female (*n* = 14) mice were used for control comparison. Two‐way ANOVA revealed a significant main effect of exercise (F(1,36) = 59.86, *p* < 0.0001) and significant main effect of sex (F(1,24) = 8.563, *p* = 0.0059) on serum ketone bodies. Gene expression analysis of ketone body metabolism in (b) Hepatic, (c) Cardiac and (d) Quadriceps muscle tissue harvested from sedentary (non‐exercised) male and female mice (*n* = 4–5 each group). *Bdh1*, beta‐hydroxybutyrate dehydrogenase 1; *Hmgcs2*, Hydroxymethylglutaryl‐CoA synthase 2; *Acat1*, acetyl‐CoA acetyltransferase 1; *Oxct1*, 3‐oxoacid CoA‐transferase 1. *p < 0.05 vs. male or female sedentary group. #*p* < 0.05 vs. male EEC group

We previously observed that endurance exercise resulted in an elevation of serum ketone body levels (Holcomb et al., 2021). To investigate potential sex‐differences in “exercise‐induced ketosis,” we analyzed ketone body concentration at the end of exercise and compared these values to sedentary mice. Both male and female mice exhibited significantly higher serum ketone bodies (both acetoacetate and 3‐hydroxybutyrate) at the end of the exercise test; however, serum ketone bodies were ~45% higher in females compared to males at the end of endurance exercise (Figure 4a). To examine whether the higher serum ketone body concentration was due to greater ketone body production, we assessed expression of genes involved in ketogenesis. In sedentary livers of male and female mice, gene expression of 3‐hydroxybutyrate dehydrogenase 1 (*Bdh1*), hydroxymethylglutaryl‐CoA synthase 2 (*Hmgcs2*), and acetyl‐CoA acetyltransferase 1 (*Acat1*) were not significantly different, suggesting no sex differences in hepatic ketogenesis. We then examined expression of genes involved in ketone body oxidation in the heart and quadriceps muscle of sedentary male and female mice. No statistically significant differences were observed for *Bdh1*, 3‐oxoacid CoA‐transferase 1 (*Oxct1*, also known as *Scot)*, or *Acat1*. Although no sex differences exist in the metabolic pathways involved in ketone body synthesis or oxidation in the heart, liver, and skeletal muscle, females exhibit elevated exercise‐induced ketosis in response to endurance exercise.

### 3.5 Markers of enhanced exogenous and endogenous lipid metabolism in female mice

**FIGURE 5:**
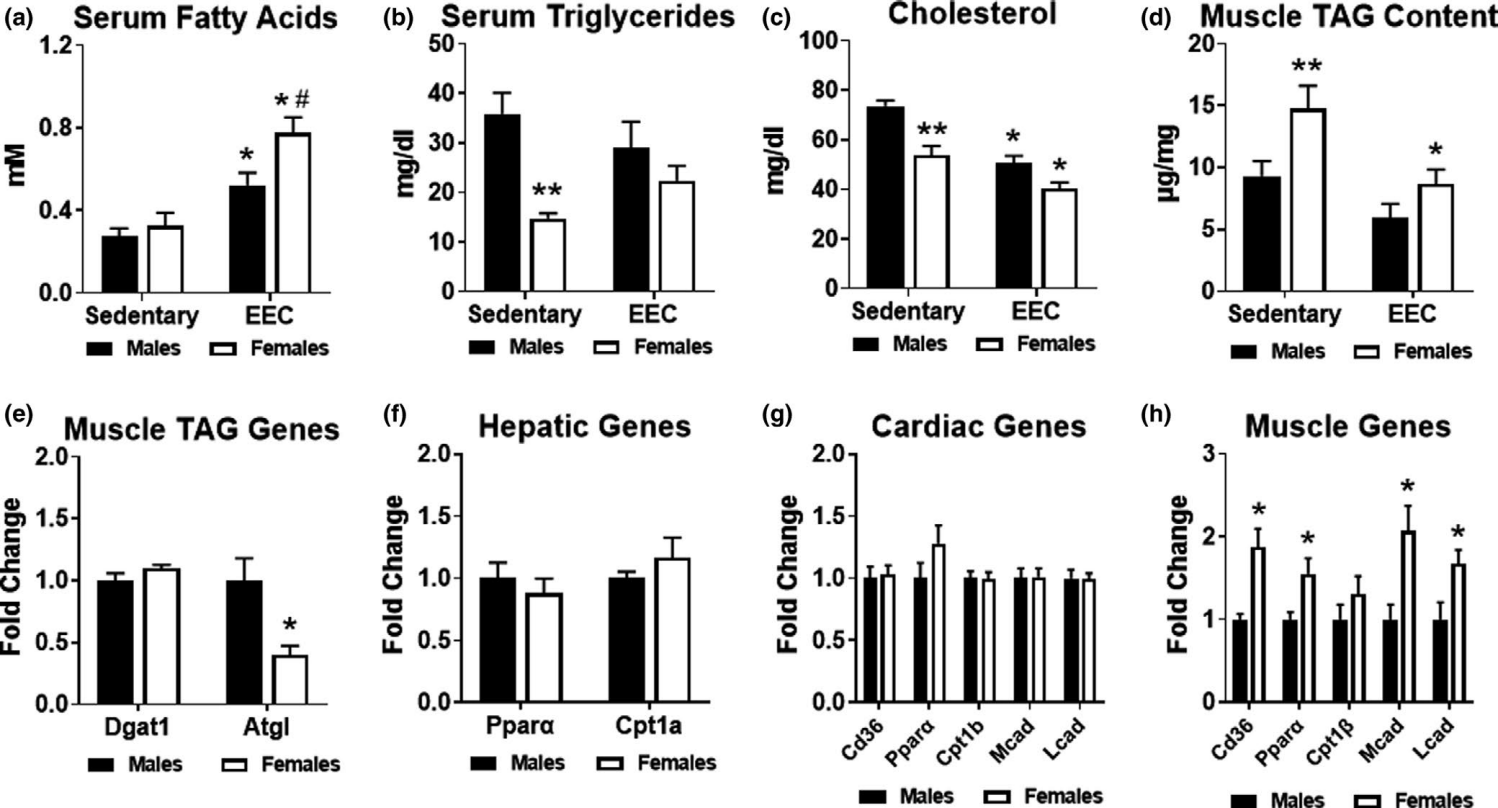
Assessment of lipid metabolism in male and female mice. Serum obtained from sedentary (non‐exercised) male (*n* = 9) and female (*n* = 12–13) mice were used for control comparisons. (a) Serum fatty acids in male and female mice at the immediate conclusion of the endurance exercise capacity (EEC) test (*n* = 9 each group). Two‐way ANOVA revealed a significant main effect of exercise (F(1,35) = 32.20, *p* < 0.0001) and significant main effect of sex (F(1,35) = 6.044, *p* = 0.0190) on serum fatty acids. (b) Serum triglycerides in male and female mice at the immediate conclusion of the endurance exercise capacity (EEC) test (*n* = 9 each group). Two‐way ANOVA revealed a significant interaction effect of exercise and sex (F(1,36) = 4.618, *p* = 0.0384) and significant main effect of sex (F(1,36) = 16.55, *p* = 0.0002) on serum triglycerides. (c) Serum cholesterol measured in male and female mice at the immediate conclusion of the endurance exercise capacity (EEC) test (*n* = 9 each group). Two‐way ANOVA revealed a significant main effect of exercise (F(1,36) = 32.02, *p* < 0.0001) and significant main effect of sex (F(1,36) = 21.93, *p* < 0.0001) on serum cholesterol. (d) Triacylglycerol (TAG) content measured in the gastrocnemius muscle obtained from sedentary and EEC male and female mice (*n* = 7 each group). Two‐way ANOVA revealed a significant main effect of exercise (F(1,24) = 11.70, *p* = 0.0022) and significant main effect of sex (F(1,24) = 8.783, *p* = 0.0068) on TAG content. (e) Analysis of genes involved in muscle TAG metabolism from quadriceps muscle tissue (*n* = 4 each group). Gene expression analysis of fatty acid oxidation in (f) Hepatic, (g) Cardiac and (h) Quadriceps muscle tissue harvested from sedentary (non‐exercised) male and female mice (*n* = 4–5 each group). *p < 0.05 male or female EEC vs. male or female sedentary group. #*p* < 0.05 vs. Male EEC group. **p < 0.05 vs. male sedentary group. *Dgat1*, diacylglycerol acyltransferase 1; *Atgl*, adipose triglyceride lipase; *Pparα*, peroxisome proliferator‐activated receptor alpha; *Cpt1a*, carnitine palmitoyltransferase 1A; *Cd36*, cluster of differentiation 36; *Cpt1b*, carnitine palmitoyltransferase 1B; *Mcad*, medium chain acyl‐CoA dehydrogenase; *Lcad*, long‐chain acyl‐CoA dehydrogenase

Since our examination of glucose and ketone body metabolism did not uncover notable sex differences, we assessed serum lipid metabolites and skeletal muscle triglyceride content. Serum fatty acids in both males and females were significantly greater in response to endurance exercise compared to respective sedentary groups (Figure 5a). Similar to serum ketone bodies, serum fatty acids were significantly greater in females at the end of exercise, consistent with higher adipose tissue lipolysis (Figure 5a). Although serum triglycerides were significantly lower in sedentary females compared to sedentary males, no significant differences were noted at the end of endurance exercise (Figure 5b). Serum cholesterol was also significantly lower in sedentary females; however, both males and females exhibited significant reductions in cholesterol in response to exercise (Figure 5c). In sedentary females, triglyceride content was nearly 50% higher in female gastrocnemius muscle (Figure 5d). At the end of the EEC, only females had a significantly lower triglyceride content compared to baseline levels (Figure 5d). In sedentary skeletal muscle, gene expression of diacylglycerol transferase 1 (*Dgat1*), the enzyme involved in the final step of triglyceride synthesis, was similar in males and females; however, adipose triglyceride lipase (*Atgl*), the enzyme involved in triglyceride lipolysis, was significantly lower in females (Figure 5e). Peroxisome proliferator activated receptor alpha (*Pparα*), the master transcriptional regulator of lipid metabolism, and carnitine palmitoyltransferase 1A (*Cpt1a*), the mitochondrial fatty acid transporter, were not statistically different in the hepatic tissue of males and females (Figure 5f). To evaluate the fatty acid oxidation pathway, we analyzed gene expression of cluster of differentiation 36 (*Cd36*), *Pparα*, carnitine palmitoyltransferase 1b (*Cpt1b*), medium‐chain acyl‐CoA dehydrogenase (*Mcad*), and long‐chain acyl‐CoA dehydrogenase (*Lcad*). As shown in Figure 5g, none of these fatty acid oxidation genes were significantly different in the hearts of males and females. However, all of these fatty acid oxidation genes, except for *Cpt1b*, were 1.5 to 2‐fold higher in in the quadriceps muscle of females (Figure 5h). Taken together, these data suggest that female mice have a greater capacity and utilization of lipids, particularly serum fatty acids and intramyocellular triglyceride stores, which may contribute to enhanced endurance exercise performance.

## 4 DISCUSSION

In the present study, we sought to identify sex differences in endurance exercise capacity and determine the potential metabolic underpinnings accounting for these differences. Our data provides several key findings. First, when subjected to exercise of long‐duration and moderate intensity, female mice can run on a motorized treadmill for nearly 2 h, which was approximately 30 min longer than male counterparts. Second, despite similar usage of exogenous glucose during exercise, female mice have higher serum fatty acids and ketone bodies, suggestive of greater mobilization of these substrates during exercise. Third, basal skeletal muscle triglyceride content is higher in female mice, which may allow for greater usage of endogenous lipids during exercise. Last, skeletal muscle of female mice has higher expression of genes involved in fatty acid uptake and oxidation, which may reflect a greater capacity to utilize fatty acids for energy. These combined findings suggest that greater mobilization of fatty acids from exogenous and endogenous sources as well as greater utilization of lipids within the skeletal muscle may account for higher endurance exercise capacity in female mice.

Sex differences in physical attributes are known, such as greater skeletal muscle mass in males and higher body fat composition in females. In addition, past research supports the notion of greater muscle strength (Miller et al., 1993) and VO_2max_ (Joyner, 1993) in males. However, muscle fatigue (also referred to as performance fatigability) is less in females than males (Hunter, 2016). Females are also reported to have a higher prevalence of Type I skeletal muscle fibers (Haizlip et al., 2015; O'Reilly et al., 2021). Given the reduced fatigability and higher Type I muscle fibers, greater exercise endurance in females would be expected. Previous studies in rodents supported the idea of higher endurance capacity in females (Konhilas et al., 2004; McMullan et al., 2016; Oydanich et al., 2019). However, some of these studies relied on total distance achieved during chronic exposure to voluntary running wheels (Konhilas et al., 2004; McMullan et al., 2016), which may be more reflective of physical activity rather than endurance capacity. Other studies that assessed exercise capacity on motorized treadmills utilized testing protocols of shorter durations of approximately 30 min (Konhilas et al., 2004; Oydanich et al., 2019). Since the utilization of lipids for energy becomes more prominent during longer durations (Egan & Zierath, 2013; Faldt et al., 2004), longer testing times might be required to adequately assess endurance capacity. To this end, we specifically selected a testing protocol that challenged exercise capacity for greater than 90 min and confirmed that female mice can exercise at moderate intensity significantly longer than male mice.

Under normal physiological conditions, the liver serves to store glycogen during periods of glucose excess and perform gluconeogenesis during periods of reduced glucose availability. When blood glucose is low, the liver stimulates glycogenolysis and gluconeogenesis to help maintain glucose homeostasis (Trefts et al., 2015). In turn, blood glucose becomes a critical exogenous substrate for the skeletal muscle for use as an immediate energy source or to be stored endogenously as glycogen. Therefore, blood glucose and muscle glycogen are important energy sources for the exercising muscle (Hoppeler, 1999). Our data show that both hepatic and muscle glycogen storage are similar between sedentary male and female mice, which is consistent with previous reports (Roepstorff et al., 2002; Roepstorff et al., 2006b). Although females are purported to have greater insulin sensitivity (Nuutila et al., 1995), this is not manifested in basal glucose levels as male and female mice are similar in our study. In response to long‐duration aerobic exercise, our data support the idea that no sex differences exist in muscle glycogenolysis or hepatic glycogenolysis and gluconeogenesis, as muscle glycogen, blood glucose, and liver glycogen are depleted to a similar extent. Interestingly, liver glycogen was nearly fully exhausted in both sexes, underscoring the duration of the exercise session and the potential reliance on other substrates, presumably lipids, to fuel exercise metabolism during this period.

Previous studies demonstrated lower respiratory exchange ratios (RERs) in females during exercise, suggestive of more lipid oxidation compared to male counterparts (McKenzie et al., 2000; Roepstorff et al., 2006b). In part, the greater lipid oxidation may be due to utilization of various sources, including endogenous triglyceride stores within the skeletal muscle as observed in previous studies (Mittendorfer et al., 2002; Roepstorff et al., 2002; Roepstorff et al., 2006a). Consistent with this, we observed higher skeletal muscle triglycerides in sedentary females, which was significantly lower at the conclusion of the endurance exercise test. In addition, we observed higher serum fatty acids concentrations in females. These combined findings suggest that females have an inherent availability of endogenous lipid sources as well as a higher capacity to mobilize exogenous lipid sources. Moreover, the significantly lower skeletal muscle triglycerides would infer a greater utilization of this source to support aerobic metabolism.

To further interrogate potential sex differences in lipid metabolism, we analyzed several genes involved in triglyceride metabolism and fatty acid oxidation within the liver, heart, and skeletal muscle of sedentary mice. Our analysis revealed no differences in several fatty acid oxidation genes in the liver and heart, indicating that transcriptional regulation of this pathway is similar between the sexes. However, we did identify several transcriptional differences within the skeletal muscle. Interestingly, we found that adipose triglyceride lipase (*Atgl*) was significantly lower in female skeletal muscle. Since *Atgl* is the primary enzyme that controls muscle lipolysis (Haemmerle et al., 2006), the reduced gene expression could account for the higher triglyceride content. With respect to the fatty acid oxidation pathway, 4 of 5 genes were higher in sedentary female skeletal muscle compared to sedentary male. *Cd36* is a membrane fatty acid transporter, which is suggested to play a key role in muscle fuel selection and endurance exercise performance (McFarlan et al., 2012). Therefore, the higher expression of *Cd36* in female mice would be suggestive of greater cellular fatty acid uptake. *Pparα* is a transcriptional regulator of lipid metabolism that regulates enzymes that control fatty acid oxidation (Muoio et al., 2002). In the present study, expression of *Pparα* was approximately 50% higher in female skeletal muscle compared to males. Not surprisingly, both *Mcad* and *Lcad*, two genes involved in beta‐oxidation, were nearly 2‐fold higher in females, which supports the notion that females have greater capacity for fatty acid oxidation in the skeletal muscle (Maher et al., 2010). We did not observe a significantly higher expression of *Cpt1b*, a rate‐limiting enzyme that controls acyl‐CoA transport into the mitochondria for beta‐oxidation (Ramsay et al., 2001). However, since *Cpt1b* regulation of fatty acid oxidation is more dependent upon malonyl CoA inhibition and sensitivity (Vavrova et al., 2016; Weeghel et al., 2018), the gene expression and protein content may not be truly indicative of the oxidative capacity.

Given the interest in the role of ketone body metabolism in exercise performance (Harvey et al., 2019) and cardiac pathologies (Kolwicz et al., 2016), we expanded our study to evaluate potential sex differences in ketogenesis and ketone body oxidation. Our findings show that endurance exercise causes elevated ketone body concentrations in the blood of both male and female mice, with significantly higher concentrations in females. Interestingly, higher ketone body concentrations after exercise were reported in the early 1900s (Koeslag, 1982), which was termed “post‐exercise ketosis” (Passmore & Johnson, 1958) and primarily referred to the exercise recovery period. The increase in circulating ketone bodies is thought to coincide with liver glycogen depletion, which can be attenuated with exercise training and carbohydrate intake (Adams & Koeslag, 1988, 1989). However, whether sex differences exist in this response or whether higher ketone body levels affect exercise performance is unclear. We recently observed that female mice fed either a chow or ketogenic diet exhibited higher serum ketone body concentrations at the end of endurance exercise (Holcomb et al., 2021). Although this “exercise‐induced ketosis” was significantly higher in the ketogenic diet fed group, exercise performance remained similar to chow fed controls (Holcomb et al., 2021). Therefore, the higher ketone body concentration in females is unlikely to be the sole contributor to the observed increased endurance exercise performance in the present study. Likewise, we observed no sex differences in the genes responsible for hepatic ketogenesis or ketone body oxidation in the heart and muscle. However, since we did not assess ketone body uptake and oxidation directly, we cannot exclude the role that sex differences in ketone body metabolism play in exercise performance.

One critique of the findings from our study is the body weight differences in males versus females. Since the EEC test was conducted on an incline, the increased body weight in males could account for the decreased endurance capacity. Our data suggest that the relative muscle mass (i.e., quadriceps mass to body weight ratio) is similar between age‐matched males and females. Although this quadriceps mass to body weight ratio is a potentially crude measurement, a recent study found that summed muscle weights were highly correlated with lean body mass in male and female mice (O'Reilly et al., 2021). Moreover, a previous study demonstrated that females had enhanced endurance capacity compared to both age‐matched and weight‐matched males (Oydanich et al., 2019). Therefore, the higher endurance exercise performance in females observed in the present study is likely due to factors beyond body weight and body composition.

There are several considerations of the experimental design of the present study that may limit the interpretation of the findings. First, although we measured beta‐estradiol concentration, we did not control for the estrus cycle in the female mice. However, a recent study concluded that the consideration of estrus cycle in studies involving exercise performance in female mice is not a limiting factor (Aguiar et al., 2018). Moreover, meta‐analyses of human studies suggest that the effect of the menstrual cycle on exercise performance in females is inconsistent and inconclusive (McNulty et al., 2020; Pereira et al., 2020). Second, the measurements of serum and tissue metabolite content was measured at the end of exercise. Although these assessments occurred at the same end point (i.e., exhaustion), the assessments were not made at the same time point of exercise, since male mice exercise nearly 30 min less. However, the intensity (ie., speed and grade of the treadmill) was similar between the male and female mice at the point of exhaustion, so any changes observed in females should be related to the increased duration. Last, the measurements of glycogen and triglyceride content or gene expression analysis was limited to the gastrocnemius and quadriceps, respectively, so the observed findings may not be reflective of changes occurring in other skeletal muscles.

In summary, the present study employed a comprehensive approach to understanding sex‐specific differences in metabolism during endurance exercise. Specifically, we evaluated glucose, lipid, and ketone body pathways in multiple tissues from both sedentary and exercised mice to uncover sex differences in exercise metabolism. Overall, the findings demonstrate that higher mobilization and utilization of lipids within the skeletal muscle are associated with greater endurance exercise capacity in female mice. Additionally, we identified elevated serum ketone body concentration in females at the end of exercise, which may indicate a greater “exercise‐induced ketosis.” We also reported lower serum triglyceride and cholesterol concentrations and higher skeletal muscle triglyceride content in sedentary female mice. The results yielded from this study provide important insight about the physiological and biochemical sex differences during endurance exercise.

## CONFLICT OF INTEREST

The authors declare no conflicts of interest.

## AUTHOR CONTRIBUTION

L.E.H. and S.C.K. conceived and designed research; L.E.H., P.R., C.C.O., E.A.D., and S.C.K. performed experiments; L.E.H., P.R., and S.C.K. analyzed data; L.E.H. and S.C.K. interpreted results of experiments; L.E.H., P.R., and S.C.K. prepared figures; L.E.H. and S.C.K. drafted manuscript; L.E.H., P.R., C.C.O., E.A.D., and S.C.K. edited and revised manuscript; L.E.H., P.R., C.C.O., E.A.D., and S.C.K. approved final version of manuscript.
